# Sustainability-based assessment using HPLC method for estimation of colchicine and dexamethasone for multi-disease treatment: application to laboratory-made combination and rat plasma

**DOI:** 10.1186/s13065-026-01723-2

**Published:** 2026-03-16

**Authors:** Aya R. Ahmed, Marwa A. A. Ragab, Mohamed A. Korany, Samar Abu khashaba, Simone A salama, Sara I. Aboras

**Affiliations:** 1 Department of Pharmaceutical Analytical Chemistry, Faculty of Pharmacy, Alexandria University, Alexandria, Egypt; 2 Department of Pharmacology and Toxicology, Faculty of Pharmacy, Alexandria University, Alexandria, Egypt

**Keywords:** Dexamethasone, Colchicine, HPLC-DAD, Rat plasma, Sustainability-based assessment, EPPI, VIGI.

## Abstract

**Objective:**

Colchicine (COL) and dexamethasone (DEX) are well-established anti-inflammatory agents with proven efficacy across a spectrum of conditions, including gout, familial Mediterranean fever, cardiovascular diseases, and COVID-19. Their combined therapeutic use offers a synergistic effect that enhances inflammation control and immune modulation. Consequently, this study aimed to develop and validate a simple, sensitive, and sustainability-oriented HPLC-DAD method for the simultaneous determination of COL and DEX in laboratory-made tablets and rat plasma, and to perform a multi-tool assessment of the method’s environmental and innovative performance.

**Methodology:**

Chromatographic separation was achieved on a reversed-phase long C8 column using acetonitrile:0.025 M phosphate buffer (pH 3) (40:60, v/v) at 1.0 mL/min using DAD tuned at 240 nm. Working ranges were 0.25–20 µg/mL (laboratory mixtures) and 1–20 µg/mL (plasma calibration with IS). Sample preparation for plasma employed protein precipitation with acetonitrile, centrifugation, evaporation, and reconstitution (300 µL). Method validation followed ICH and FDA bioanalytical guidance.

**Key findings:**

The proposed method enabled the rapid and efficient separation of COL and DEX, yielding well-resolved peaks within 5 min and an excellent chromatographic performance. The method exhibited outstanding linearity (*r* > 0.999), high sensitivity, and reliable precision (intra- and inter-day RSD ≤ 6.25%), with extraction recoveries exceeding 85% in plasma. The sustainability assessment confirmed the environmentally friendly nature of the method, achieving high scores using multiple assessment metrics (Analytical Eco-Scale = 86; AGREE = 0.76; BAGI = 72.5; RGB model whiteness = 91.7). In addition, the violet innovation grade index (VIGI = 55) indicated a notable level of innovation, and the Environmental, Performance, and Practicality Index (EPPI = 87) indicated a high level of environmental sustainability, analytical efficiency, and real-world applicability.

**Impact:**

This is the first reported chromatographic method enabling simultaneous quantification of COL and DEX in pharmaceutical and plasma matrices together with a comprehensive, multi-tool sustainability assessment (including VIGI and EPPI). The method’s short run time, high sensitivity, and favorable sustainability scores support its application in routine quality control and preclinical pharmacokinetic studies.

**Graphical Abstract:**

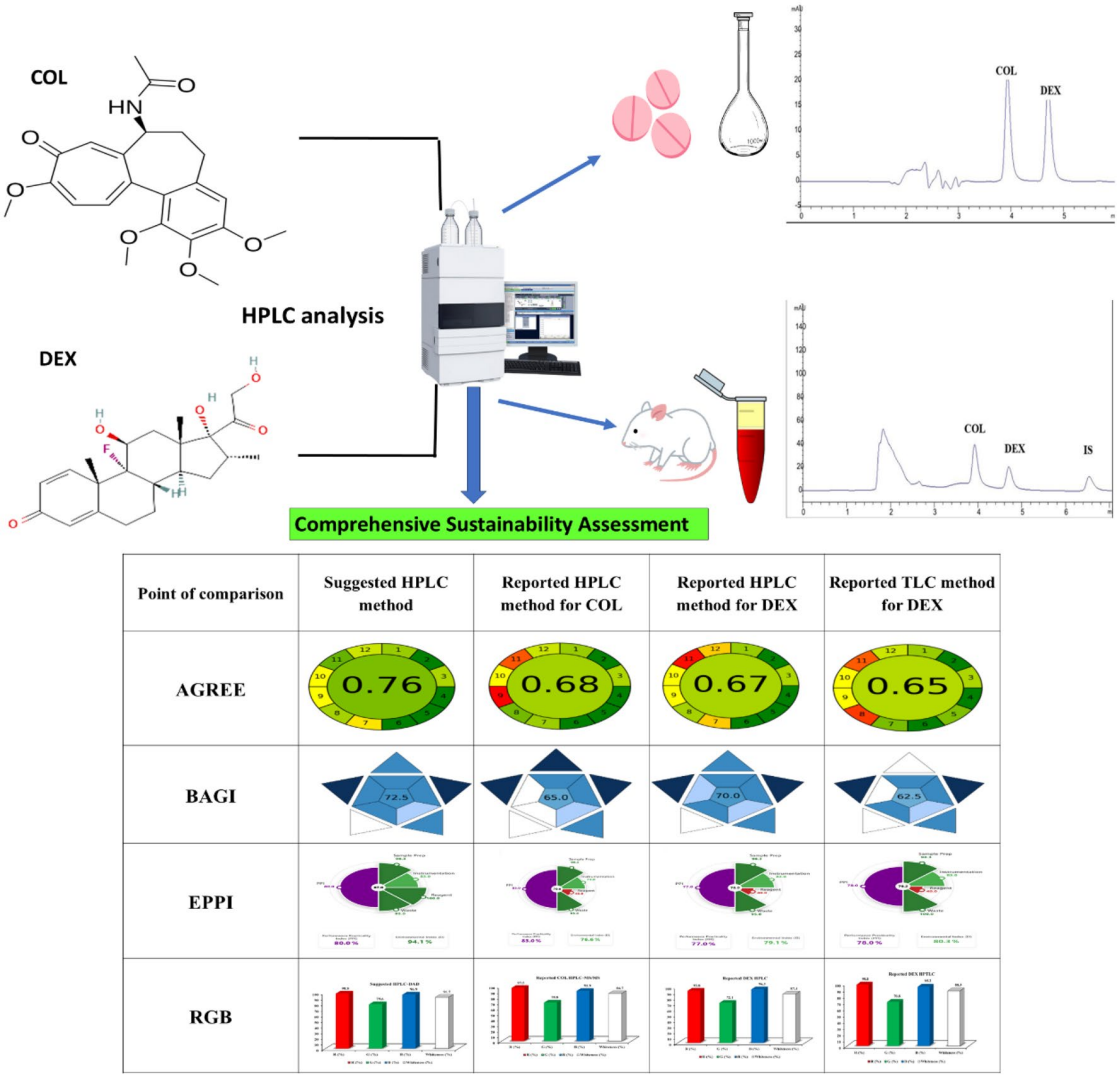

**Supplementary Information:**

The online version contains supplementary material available at 10.1186/s13065-026-01723-2.

## Introduction

**Fig. 1:**
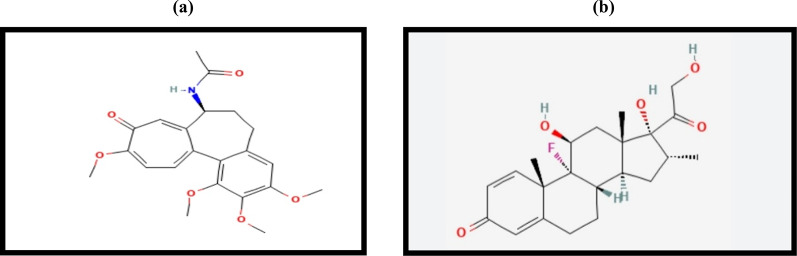
Structures of Colchicine (**a**) and Dexamethasone (**b**)

Colchicine (COL), is chemically known as N-[(7 S)-1,2,3,10-tetramethoxy-9-oxo-6,7-dihydro-5 H-benzo[a]heptalen-7-yl] acetamide, Fig. 1a [1]. It is widely recognized for its potent anti-inflammatory effects, making it a valuable treatment option for various conditions: gouty arthritis and familial Mediterranean fever [2]. Furthermore, inflammation promotes atherosclerosis and thrombosis, increasing strokes, making COL a promising therapy for high-risk cardiovascular patients [3].

Dexamethasone (DEX), Fig. 1b, is a synthetic glucocorticoid called 9α-fluoro-11β,17α,21-trihydroxy-16α-methylpregna-1,4-diene-3,20-dione [4]. It provides anti-inflammatory effects via neutrophil inhibition and lymphocyte suppression and is often used with antibiotics for immune-related conditions [5]. It is classified as an essential medicine and is recommended by the National Institute of Health in the United States and the National Health Service in the United Kingdom for COVID-19 patients needing mechanical ventilation or oxygen therapy [6].

Combination therapy can enhance efficacy by targeting multiple inflammatory pathways. The COL–DEX pairing provides complementary anti-inflammatory and immunomodulatory actions, demonstrating reductions in inflammation, late-stage edema, and hospital mortality in COVID-19 patients [7, 8]. Emerging formulations, such as dual-release microneedle patches, further highlight growing interest in this pair [9].

The therapeutic relevance of this synergistic combination in inflammatory conditions motivated the development of an analytical method for the accurate simultaneous determination of both drugs in pharmaceutical dosage forms and biological fluids. A review of the literature revealed the absence of a validated HPLC method for their concurrent analysis, representing a clear research gap. The present study addresses this gap by introducing a novel HPLC approach and evaluating its sustainability using different assessment tools, thereby supporting future pharmacokinetic studies and optimized treatment strategies.

The determination of COL in pharmaceutical formulations and biological fluids, both alone and in combination with other medications, has been the subject of several investigations. Numerous analytical methods have been used, such as spectrophotometry [10, 11], HPLC in pharmaceutical dosage forms [12, 13], HPLC in biological fluids [12, 14, 15], HPTLC [16, 17], spectrofluorimetry [18], alongside electrochemical [19] and capillary electrophoresis methods [20].

Similarly, DEX has been analyzed both individually and in combination with other pharmaceuticals using different analytical approaches across various matrices. These include spectrophotometric methods [21, 22], HPLC in biological fluid [23, 24], HPLC in pharmaceutical formulations [25, 26], HPTLC [27–29], voltammetry [30], and capillary electrophoresis [31].

No chromatographic or bioanalytical method has previously been reported for the simultaneous determination of COL and DEX in pharmaceutical products or biological matrices. Given the clinical relevance of their co-administration, reliable in vivo quantification is essential. In this work, we developed a simple, green, accurate, and sensitive chromatographic method for the concurrent determination of COL and DEX in laboratory-prepared mixtures and in rat plasma following different routes of administration. This provides a foundational platform for future pharmacokinetic and clinical investigations on this emerging drug combination. The method’s greenness and sustainability were further evaluated using multiple assessment tools, including the Analytical Eco-Scale, AGREE, RGB12, BAGI, EPPI and VIGI, supporting the development of more innovative and sustainable analytical practices [32–37].

## Experimental

### Instrumentation

A computer running Agilent ChemStation Software (Agilent Technologies, Santa Clara, CA, USA) was linked to an Agilent 1200 series HPLC-DAD system, which contained a diode array detector (DAD), vacuum degasser, quaternary pump, and auto-injector. A J.P. SELECTA, S.A. sonicator from Abrera (Barcelona, Spain) was utilized for sample preparation. pH measurements were conducted using a pH meter, Crison - Model Basic 20, manufactured by Crison Instruments, S.A. (Barcelona, Spain). A precision balance KERN AEJ 220-4 M (Germany) was employed for weighing, while centrifugation was performed using a HerMle centrifuge, Type: Z206A (Germany). Additionally, the organic layer was evaporated to dryness under vacuum conditions using a Christ rotational vacuum concentrator (RVC 2–18 CD plus, Germany).

###  Materials

COL (99.5%) was obtained from Pharaonia Pharmaceuticals Company, Alexandria, Egypt, while DEX (98.9%) and domperidone (99.3%), the internal standard (IS), were supplied by Pharco Pharmaceuticals Company, Alexandria, Egypt. Acetonitrile and HPLC-grade methanol were obtained from Fisher Scientific (Loughborough, UK). Commercial pharmaceutical products, *Colchicine®* (0.5 mg per tablet) and *Dexamethasone®* (2 mg per tablet), were purchased from the local market. The *Colchicine®* tablets (0.5 mg) contained microcrystalline cellulose, lactose monohydrate, sodium starch glycolate, magnesium stearate, and povidone as excipients, while *Dexamethasone®* tablets (2 mg) comprised calcium phosphate, lactose, magnesium stearate, and starch as inactive ingredients. Orthophosphoric acid, sodium dihydrogen phosphate monohydrate, and sodium hydroxide were sourced from Sigma-Aldrich Chemie GmbH, Switzerland, Germany.

### Ethics approval and consent to participate

Six healthy adult male Wistar rats (180–220 g) were obtained from the accredited Animal House of the Faculty of Pharmacy, Alexandria University, Alexandria, Egypt. The animals were housed and maintained in the same facility under standard laboratory conditions and were used as the source of biological fluids in this study.

The study protocol was reviewed and approved by the Ethics Committee of the Faculty of Pharmacy, Alexandria University, Alexandria, Egypt (Ethical approval number: AU06202412242258). All procedures were conducted in accordance with the ARRIVE guidelines and relevant animal welfare regulations.

### Standard solutions preparation

Standard stock solutions comprising 500 and 1000 µg/mL of COL and DEX, respectively, were made in HPLC-grade methanol. In addition, the Internal standard (IS) stock solution was 400 µg/mL, and its working stock solution was 40 µg/mL for the suggested chromatographic technique. For a maximum of one week, these stock solutions were kept in a refrigerator at 4 °C. For each medication’s analysis, working stock solutions were also made in methanol (HPLC-grade) at a concentration of 100 µg/mL. Distilled water was used to dilute several aliquots of the working stock solutions to achieve the targeted concentration ranges of 0.25–20 µg/mL for both COL and DEX. Furthermore, samples for quality control (QC) were created for further analysis.

### Preparing the calibration and quality control samples for the in vivo analysis

100 µL of drug-free rat plasma was spiked with varying aliquots of the working standard solutions of the COL and DEX, each at 100 µg/mL, to create calibration standards and QC samples. A final concentration of 12 µg/mL of the IS was added. The samples were mixed with 2 mL of acetonitrile for drug extraction and protein precipitation. After two minutes of high-speed vortex mixing, the tubes were centrifuged for ten minutes at 5000 g. A vacuum concentrator was used to thoroughly evaporate the organic layer until it was completely dry. The residues were reconstituted using 300 µL of acetonitrile to achieve final concentrations within the linearity range. A single set of standards and QC samples was examined every working day using the predetermined technique.

The calibration standards were made at concentrations of 1, 3, 5, 10, 15, and 20 µg/mL for both COL and DEX. Lower limit of quantitation (LLOQ), low quality control (LQC), medium quality control (MQC), and high-quality control (HQC) were the four QC samples that were prepared at various levels: 1, 3, 10, and 15 µg/mL for the cited medications.

### Chromatographic conditions

Acetonitrile and 0.025 M phosphate buffer (pH 3), which was made by modifying sodium dihydrogen phosphate monohydrate with ortho-phosphoric acid, in a (40%:60%, v/v) ratio, respectively, made up the mobile phase. The buffer was filtered through a 0.45 μm Millipore filter before use. A steady flow rate of 1 mL/minute was maintained during the study. A 20 µL injection volume was used. To ensure high sensitivity for both drugs, they were detected using DAD tuned at 240 nm, which corresponds to the λ_max_. All analyses were carried out at room temperature.

### Assay of the studied medications in laboratory-made combinations and in rat plasma

COL and DEX represent a novel combination regimen for multi-disease treatment. They are co-administered with various ratios, for example, they are taken in ratios 1:5 (COL: DEX) for reducing inflammation and late-stage edema in experimental brain contusions [7], in addition to patients with COVID-19 pneumonia treated with COL and corticosteroids in both 10:10 and 2:1 ratios [8, 38]. Besides patients with COVID-19 pneumonia and acute respiratory distress syndrome, they are co-administered in a 1:10 ratio [39].

#### In laboratory-made combinations

After being precisely weighed, ten *Colchicine*® tablets were crushed into a fine powder. A 10-mL volumetric flask was filled with a part of the powdered mass corresponding to 5 mg of COL. The sample was then dissolved in methanol to create the stock solution. To guarantee total dissolution, the solution was sonicated for 20 min. Following sonication, methanol was used to regulate the volume, and Whatman No. 1 filter paper was used for filtering.

Ten *Dexamethasone*® pills were also weighed and ground into a fine powder. In order to create the stock solution, 10 mg of DEX was placed into a 10-mL volumetric flask and dissolved in methanol. The final stock solution concentrations were 500 µg/mL for COL and 1000 µg/mL for DEX.

To achieve a final concentration of 100 µg/mL for both medications, the stock solutions were further diluted. After that, aliquots of the diluted solutions were put into 10-mL volumetric flasks and filled with distilled water. This produced four lab-made mixes with COL: DEX ratios of 1:5, 2:1, 1:10, and 10:10, respectively. Both medications were analyzed in their final dose form dilutions using these combinations, which were made within the linear concentration ranges.

#### In rat plasma

The bioanalysis of COL and DEX in male rats after oral and intraperitoneal (IP) injection was the main focus of this investigation. The rats were acclimated for three days in a controlled breeding environment with a relative humidity of 45% to 55% and a temperature of 25 ± 2 °C before the experiment started. They have constant access to the typical food and water used in laboratories.

Two sets of six male Wistar rats, weighing 180–220 g, were chosen. In one group, COL and DEX were administered orally, whereas in the other, the cited medications were injected intraperitoneally. Whereas the IP doses were made as a filtered solution after medication extraction, the oral doses were given by oral gavage as an aqueous solution. The IP group received 0.8 mg/kg of COL and 8 mg/kg of DEX, whereas the oral group received 2 mg/kg of COL and 10 mg/kg of DEX.

The rats were anaesthetized using thiopental (50 mg/kg, IP) before sample withdrawal. Rats had their blood drawn from the retro-orbital plexus at intervals of 0, 5, and 10 min for IP-treated rats, whereas rats given oral dosages had their blood drawn at 0, 1, 2, and 3 h. Euthanasia was performed by administration of a high dose of thiopental. The samples were collected in polypropylene tubes containing K₃-EDTA to prevent clotting. The blood samples were centrifuged at 5000 g for 10 min, and the separated plasma was collected and frozen at − 20 °C until analysis. On the day of analysis, the plasma samples were thawed at room temperature and vortexed for two minutes before further processing.

## Results and discussion

Given the significance of combining these medications to mitigate inflammation and edema following brain contusion, as well as their potential applications in COVID-19 treatment and hypertrophic scar therapy, this study aimed to optimize their quantification using an HPLC-DAD method. The method development was tailored to achieve optimal separation, maximum sensitivity, and minimal baseline noise. Additionally, the analysis was conducted not only on their proposed laboratory-made combination but also on rat plasma samples, with the study performed on six rats.

### Chromatographic method development and optimization

#### Choice of chromatographic column

Several reversed-phase columns, such as Zorbax SB-C8 (220 × 4.6 mm, 5 μm particle size), Zorbax C18 (150 × 4.6 mm, 3.5 μm particle size), and Zorbax C18 (250 × 4.6 mm, 5 μm particle size), were investigated in the initial experiments. Actually, excessive retention of COL and DEX, 8.2 & 10.1 min, respectively, and increased tailing (> 1.2) reduced sensitivity and increased solvent consumption when using Zorbax C18 (250 × 4.6 mm, 5 μm particle size). On the other hand, Zorbax C18 (150 × 4.6 mm, 3.5 μm particle size) resulted in rapid elution, with solvent front, especially for COL (COL, DEX; 1.7, 3.3 min). Therefore, the Long C8 column offered the best compromise between peak separation and resolution while keeping a suitable retention time. Consequently, the Long C8 column was chosen as the best choice for the suggested chromatographic technique.

#### Buffer adjustment

The retention time and peak symmetry of the target medications were not significantly affected by changing the phosphate buffer’s ionic strength (0.025, 0.05, and 0.1 M). Consequently, 0.025 M was selected for the investigation.

Various pH levels [3–9] were tried in conjunction with the mobile phase (40% acetonitrile: 60% buffer, v/v) to optimize the pH of the 0.025 M sodium dihydrogen phosphate buffer, taking into account the pKa values of the medications (COL: 12.8, DEX: 1.89 and 6.18). The findings showed that pH 3 was the ideal value since higher pH values (7 and 9) resulted in a lesser peak area and delayed elution, whereas pH 3 generated the best chromatographic performance in terms of response and retention time.

#### Effect of organic modifier

Regarding the organic modifier, first of all, we started using methanol, but it led to late elution of both drugs with a low value of peak area and broader peaks with tailing exceeding 1.5. To address this issue, acetonitrile was explored as an alternative. Various acetonitrile ratios were evaluated to determine the optimal composition for achieving proper separation and reasonable retention times. A 50% acetonitrile concentration resulted in early elution of COL (2.3 min), causing it to overlap with the solvent front. Conversely, reducing acetonitrile to 30% caused delayed elution (6.5 min for COL and 10.02 min for DEX). The optimal balance was achieved with a 40% acetonitrile ratio in the mobile phase, providing sharp peaks, good resolution, peak symmetry, and suitable retention times for both medications (3.9 min for COL and 4.7 min for DEX), as illustrated in Figure S1.

#### Wavelength selection

**Fig. 2:**
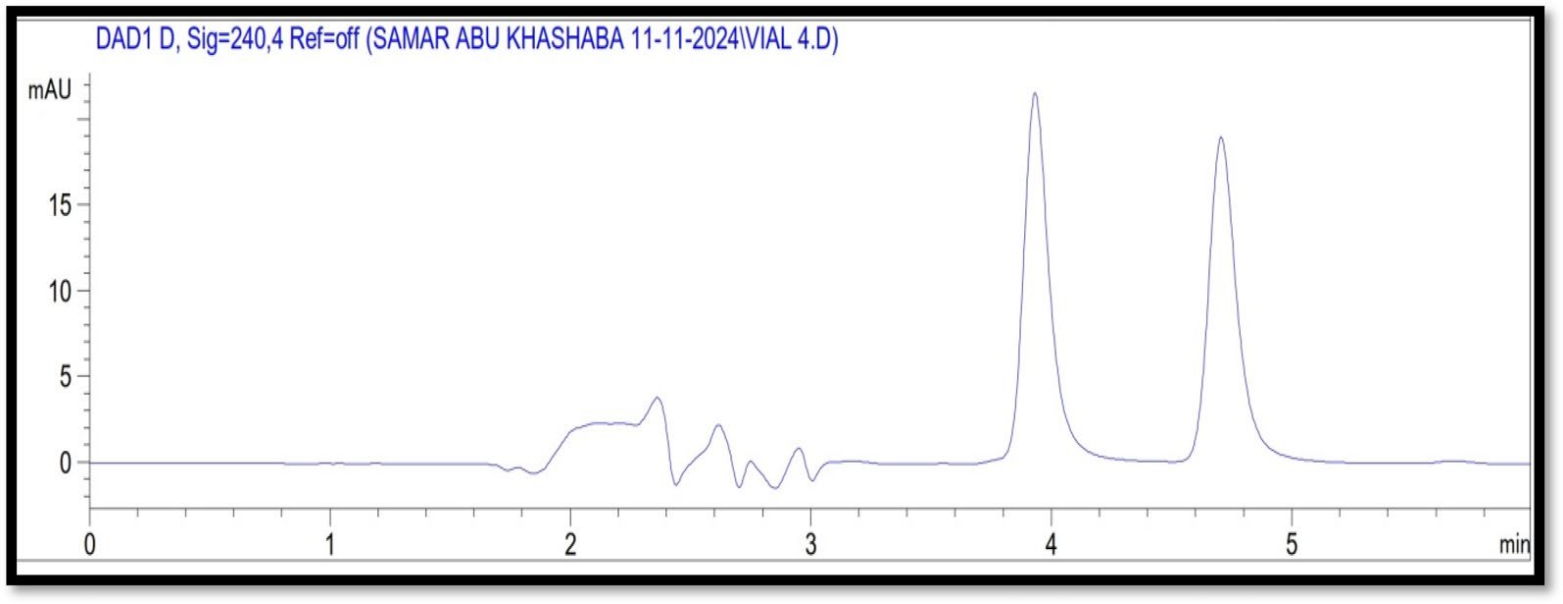
A typical HPLC chromatogram with R_t_ values of 3.9 and 4.7 min for a solution containing 2 µg/mL of COL and DEX (**a**)

To determine the most suitable wavelength for the quantification of both COL and DEX, an extensive evaluation was conducted to identify the optimal scanning wavelength that provided maximum and reliable responses for the target medications. Based on the findings, DAD was set at 240 nm (λ_max_), as this wavelength ensured higher sensitivity for the accurate determination of both medications (Fig. 2).

#### Selection of IS

Several IS were investigated for the in vivo application of chromatographic procedures: valsartan, naproxen, febuxostat, domperidone, and prednisone. Domperidone was chosen as it gave reasonable retention time, good resolution, reasonable response, and peak shape under the selected chromatographic conditions. Furthermore, it was extracted with high recovery utilizing the same extraction procedure.

#### System suitability

To guarantee optimal performance, the system suitability characteristics were assessed as part of the method development process. The capacity factor values for COL and DEX were 1 and 1.8, and the theoretical plates were 7105 and 8468, respectively. Furthermore, the resolution value between the two peaks was 3.93, and the tailing factors for COL and DEX were 0.87 and 0.89, respectively. Every parameter was within the permissible range as per the recommendations set out by USP [40, 41]. Figure 2 illustrates the successful separation of COL and DEX at 3.9 and 4.7 min, respectively, with distinct peaks, strong resolution, and outstanding peak symmetry.

### Sample extraction procedures for the in-vivo analysis

**Fig. 3:**
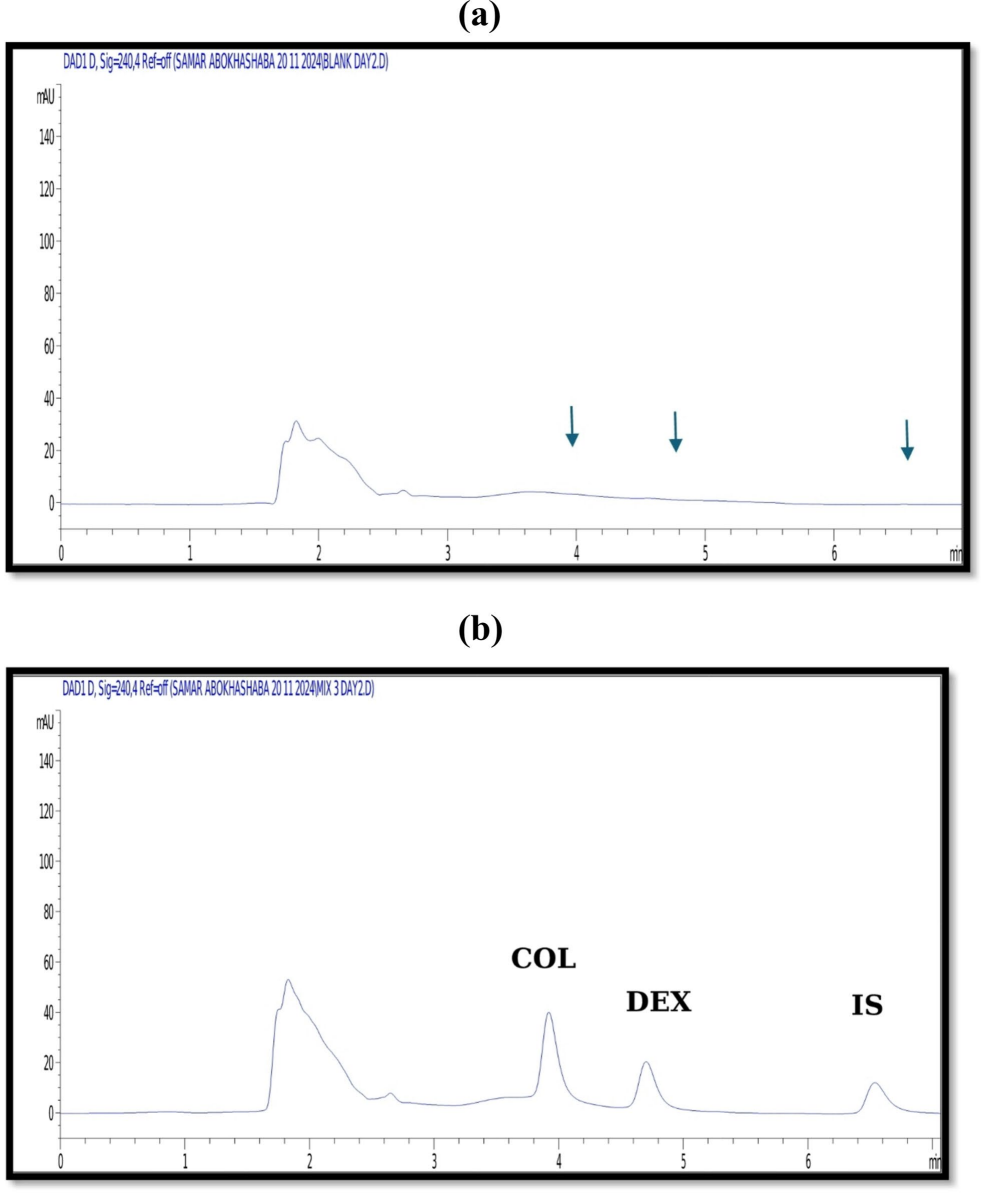
HPLC chromatograms (a) a blank plasma and (b) a blank plasma spiked with 5 µg/mL of COL, 5 µg/mL DEX, and 12 µg/mL of IS. The arrows in the blank chromatogram indicate that endogenous plasma components do not interfere with the analyte peaks (**a**)

To achieve maximum sample extraction recovery, various methods have been reported for extracting the selected medications [15, 23, 24]. Different solvents were used, including acetonitrile, ethyl acetate, and diethyl ether. While liquid–liquid extraction is known for providing excellent sample purification, protein precipitation is often preferred due to its speed, cost-effectiveness, and simplicity [15, 42, 43]. Ultimately, the choice of solvent was based on its ability to yield higher extraction efficiency with minimal matrix effects [44]. However, the best results were achieved using 2 mL of acetonitrile, followed by centrifugation and vortex mixing for 2 min, as shown in Figure S2. After that, the supernatant was concentrated at 40 °C in a vacuum concentrator. Lastly, 300 µL of acetonitrile was used for reconstitution, guaranteeing efficient separation and excellent analytical performance with good recovery, symmetrical, sharp peaks Fig. 3.

### Method validation

ICH guidelines [45] and the FDA’s 2018 Industry Guidance on Bioanalytical Method Validation [46] were followed in the method’s validation.

#### Validation of laboratory-made combinations, according to ICH [45]

##### Linearity and concentration ranges

The suggested chromatographic method’s linearity was evaluated by analyzing a range of distinct medication concentrations. To get the relevant linear regression equations, the calibration data were subjected to least squares regression analysis. The observed peak regions showed a strong relation to the COL and DEX concentrations under optimal circumstances. As shown in Table 1, the approach showed remarkable linearity, as evidenced by a high correlation coefficient (r) and low standard error of residuals (S_y/x_).

**Table 1:** Regression and statistical parameters for the suggested HPLC-DAD method for COL and DEX determination

Parameter	COL	DEX
*Linearity range	0.25-20	0.25-20
a^a^	4.09	3.72
Sa^b^	1.49	0.76
b^c^	73.40	57.33
Sb^d^	0.17	0.09
S_y/x_^e^	2.79	1.41
r^f^	0.9997	0.9998
LOD^g^	0.06	0.07
LOQ^h^	0.25	0.25

*Linearity range is in µg/mL

^a^ Intercept. ^b^ Standard deviation of the intercept

^c^ Slope. ^d^ Standard deviation of the slope

^e^ Standard deviation of residuals. ^f^ Correlation coefficient

^g^ LOD = Limit of detection (µg/mL). ^h^ LOQ = Limit of quantitation (µg/mL)

##### Detection and quantification limits

The medication concentrations that matched a signal-to-noise ratio of 3:1 for limits of detection (LOD) and 10:1 for limits of quantitation (LOQ) were identified in order to experimentally ascertain the LOD and LOQ. Practical testing was used to confirm these amounts.

While the LOQ value was 0.25 µg/mL for both COL and DEX, the technique produced low LOD values of 0.06 µg/mL for COL and 0.07 µg/mL for DEX, as shown in Table 1. These findings show that the suggested approach ensures a strong analytical response for both medications with little background noise.

##### Accuracy and precision

A wide variety of concentration ratios (0.5:5, 10:10, and 5:0.5) were used to assess the accuracy of the proposed method for the simultaneous determination of the two drugs under study. Percent error (% Er) and % recovery were computed to evaluate the accuracy of the approach. The findings showed outstanding recovery values, regularly falling between 98% and 102%, with a low %Er < 2%, supporting the method’s high accuracy. Repeatability and inter-day precision measures were also used to evaluate precision. Strong consistency between test results was shown by the relative standard deviation percentages (RSD%) for both tests being below 2%. This demonstrates the suggested method’s great precision, which is described in Table 2.

**Table 2:** Evaluation of accuracy and precision for the determination of COL and DEX using the suggested HPLC-DAD method

Drug	Concentration (µg/mL)	Accuracy		Precision	
		Mean % recovery *±* SD ^a^	E_*r*_% ^b^	Intra-day RSD%^c^	Inter-day RSD%^c^
COL	0.5 10 5	99.87 *±* 0.01 99.13 *±* 0.10 99.85 *±* 0.09	-0.13 -0.87 -0.15	1.29 0.76 0.96	1.07 0.96 0.53
DEX	5 10 0.5	100.19 *±* 0.07 99.82 *±* 0.88 99.47 *±* 0.01	0.19 -0.18 -0.53	1.12 1.09 0.71	0.82 0.94 0.60

*The ratios of the synthetic mixtures are 0.5:5, 10:10, 5:0.5 of COL and DEX, respectively

^a^Mean recovery of the found concentration ± standard deviation for three determinations (*n* = 3)

^b^% Relative error

^c^% Relative standard deviation

##### Robustness

Various chromatographic conditions were slightly altered to validate the method’s robustness as working wavelength (± 1 nm), ratio of mobile phase (± 2%), and pH of buffer (± 0.2 pH units). The effect of these changes on the peak area and the retention time values was calculated. The % standard deviation of the peak areas of each medication was estimated (COL: DEX, 0.06:0.04, respectively). The low readings for RSD% (< 2%) in company with unchanged retention times values (COL: DEX, 3.8, 4.9 min, respectively). The results obtained after making tiny deliberate adjustments to the method parameters demonstrated the robustness of the suggested method.

##### Specificity

Making sure that there was no interference from widely used excipients and additives in the laboratory-made combinations allowed for the evaluation of the method’s specificity. With a resolution value of 3.93, the assay section showed that the COL and DEX peaks were well separated, indicating successful separation. Furthermore, the analysis of laboratory-made combinations showed no additional peaks that could be attributed to excipients, indicating no interference from formulation components. The purity profiles obtained further confirmed the integrity of COL and DEX peaks, reinforcing the method’s reliability in laboratory-made combination analysis, as illustrated in Figure S3.

##### Stability of solutions

After being kept at room temperature for up to six hours, the final working standard solutions of COL and DEX showed no chromatographic changes. Furthermore, when kept in a refrigerator at 4 °C, the working and standard stock solutions of the medications under study were stable for at least a week. The stability of the solutions under the given conditions was demonstrated by the fact that no discernible deterioration occurred over this time and that the medications’ retention times and peak areas stayed constant.

#### Validation of the in-vivo rat plasma analysis, according to the FDA’s guidance [46]

##### Linearity

Using spiked rat plasma samples, calibration curves were created for the medications under analysis using the IS approach. These curves covered the whole concentration range, including the LLOQ. For both medications, the calibration curves showed linear behavior in the concentration ranges of 1–20 µg/mL. Strong linearity was verified by regression and statistical parameters, as shown in Table 3, with correlation coefficient values greater than 0.999. High F-values further confirmed the linearity of the approach. Low LLOQ showed that the technique has enough sensitivity for accurate quantification.

**Table 3:** Regression and statistical parameters for COL and DEX measurement in spiked rat plasma samples using the suggested HPLC-DAD method

Parameter	COL	DEX
Linearity range	1–20	1–20
a ^a^	-0.04	0.003
S_a_^b^	0.07	0.03
b ^c^	0.58	0.25
S_b_^d^	0.01	0.003
S _y/x_^e^	0.12	0.06
r ^f^	0.9992	0.9991
F ^g^	7862	6121
Significance F	3.46 × 10^− 9^	6.46 × 10^− 9^

*Linearity range is in µg/mL

^a^Intercept

^b^Standard deviation of the intercept

^c^Slope

^d^Standard deviation of the slope

^e^Standard deviation of residuals

^f^Correlation coefficient

^g^Variance ratio, equals the mean of squares due to regression divided by the mean of squares about regression (due to residuals)

##### Precision and accuracy

Six QC samples were made by spiking different amounts of the medications under study, including their LLOQ, at four distinct levels (LLOQ, LQC, MQC, and HQC). The accuracy and precision of the approach were then assessed by analyzing these samples. The accuracy findings, together with the inter-day and intra-day precision, are summarized in Table 4. The great precision and accuracy of the proposed approach were proved by the consistently low RSD% values (≤ 6.25) for both inter-day and intra-day precision and low % Er, respectively.

**Table 4:** Accuracy and Intra-day and inter-day precision for the determination of COL and DEX in spiked rat plasma samples using the suggested HPLC-DAD method

Drug	Level	Concentration (µg/mL)	Accuracy		Precision	
			Mean % recovery ^a^	E_r_% ^b^	Intra-day RSD%^c^	Inter-day RSD%^c^
COL	LLOQ	1	94.08	-5.92	2.98	2.29
	LQC	3	105.21	5.21	3.89	5.05
	MQC	10	100.81	0.81	6.25	3.71
	HQC	15	99.08	-0.92	1.02	2.02
DEX	LLOQ	1	104.02	4.02	3.06	5.01
	LQC	3	97.01	-2.99	5.09	2.87
	MQC	10	100.19	0.19	3.21	3.49
	HQC	15	103.51	3.51	4.56	4.01

^a^ Mean percent recovery for six determinations (*n* = 6)

^b^ % Relative error

^c^ %Relative standard deviation

##### Matrix effect

As shown in Figure S4, the same protein precipitation procedure was applied to six blank rat plasma samples to evaluate the method’s selectivity and to ensure that endogenous components did not interfere with the peaks of the target drugs. The purity of the COL, DEX, and IS peaks was verified. Since no interference from natural plasma components or medication metabolites was found, the peak purity analysis findings showed the excellent reliability of the suggested approach.

##### Dilution integrity test

This test was performed to take into consideration the potential for certain samples to surpass the maximum calibrated concentration. Higher amounts of both medications than the upper limit of quantitation (ULOQ) were added to rat plasma samples. After that, blank plasma samples were used to dilute these samples four times. Five iterations of the process were conducted prior to analysis. The findings confirmed the method’s reliability for managing high-concentration samples by showing that for each medication, the %RSD and % Er stayed within the permissible range (less than 15%).

##### Extraction recovery

By comparing the responses of the medications under study in spiked plasma samples that were treated prior to analysis with those in blank plasma samples that were processed first and then spiked with the medications, the extraction recovery was ascertained. The obtained recovery values, which were more than 85%, showed that the suggested approach had a high extraction efficiency for evaluating the targeted medications in rat plasma.

##### Carryover

To assess any residual presence of the medications in the analytical instrument, blank plasma samples were injected immediately after the ULOQ sample. The analysis confirmed that carryover in the blank samples remained below 20% of the LLOQ for both targeted medications, ensuring minimal residual interference.

##### Incurred sample reanalysis

To confirm technique reproducibility, a selection of treated rat plasma samples from several research runs was subjected to incurred sample reanalysis. For COL and DEX, six samples (10% of the total) underwent further analysis. The average recoveries ± SD were 108.11 ± 6.05 and 94.63 ± 9.32 for COL and DEX, respectively. These findings met the predetermined regulatory acceptance standards, which call for at least 70% of the reanalyzed samples to have variances of less than 20%.

##### Studies of stability

As shown in Table S1, stability tests were performed to determine how stable the examined medications were in rat plasma samples at LQC and HQC levels (*n* = 6) under varied storage conditions. The RSD% values stayed below 7.71%, while the recovered values varied from 90.04% to 108.23%, indicating that the medications under study are stable in rat plasma under ideal circumstances.

### Assay of the studied medications in laboratory-made combinations, and in rat plasma

#### In Laboratory-made combinations

The determination of COL and DEX in laboratory-prepared combinations was successfully achieved using the well-established chromatographic method. As shown previously in Figure S3, the medications were eluted at their specified retention times free from excipient interference. The percentage recovery, SD, RSD%, and %Er data, which are shown in detail in Table S2, show that the test findings were highly accurate and precise.

#### For rat plasma

**Fig. 4:**
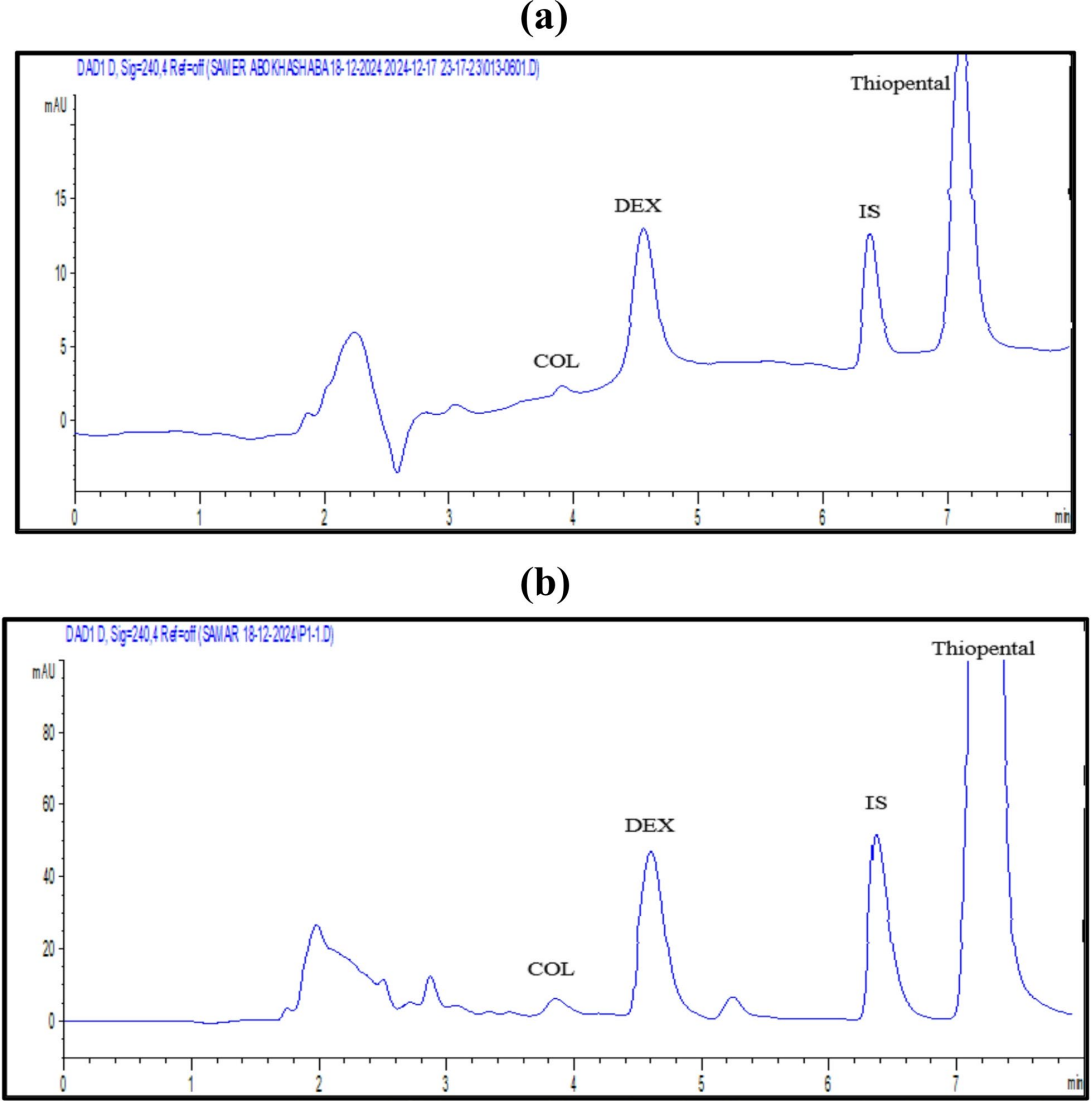
HPLC chromatograms of (a) rat plasma sample obtained after 3 h of oral administration of COL when co-administered with DEX, (b) rat plasma sample obtained after 5 min of intraperitoneal administration of COL when co-administered with DEX

The suggested approach worked well for the in vivo investigation (oral and IP) as well as for spiked samples. Both oral and IP administration routes were included to demonstrate the applicability and robustness of the developed method under different physiological conditions: a clinically relevant oral route and highly systemic IP exposure, ensuring and enabling us to test the method’s ability to detect the analytes under conditions of enhanced plasma levels. The objective was not to perform a full Pharmacokinetic study but to validate that the developed HPLC method could accurately quantify COL and DEX in plasma samples obtained under distinct pharmacological scenarios. This dual-route validation strengthens the translational relevance of the method. Rats were given a single oral dosage of 2 and 10 mg/kg and an IP dose of 0.8 and 8 mg/kg of COL and DEX, respectively, then plasma samples containing COL and DEX were withdrawn at the specified intervals. Accurate measurements were made of the medication concentrations in plasma samples obtained from each rat’s blood. The method demonstrated excellent sensitivity, recovery, and minimal matrix effects when applied to plasma samples. Representative chromatograms are shown in Fig. 4, depicting plasma concentrations five minutes post-IP injection and three hours post-oral administration. In both chromatograms, a peak appeared at 7.2 min, whose UV spectrum is similar to that of thiopental, the anesthetic drug used to calm rats during injection and blood withdrawal, ensuring the selectivity of our method for the selected drugs. The results underscore the method’s suitability for pharmacokinetic and preclinical studies involving COL-DEX combination therapy.

### The RGB model and WAC scores

Environmental and green methods have received a lot of attention lately in a variety of analytical techniques, such as spectroscopy [47, 48] chromatography [49–58] and capillary electrophoresis [59, 60]. Analytical techniques must align with environmental safety regulations and support sustainable development objectives.

A comparison with previously published HPLC and HPTLC methods for COL and DEX analysis individually was done to examine the suggested method’s sustainability, practicality, and environmental effect [15, 24, 29]. To evaluate greenness, economic feasibility, and practical application, a variety of assessment methodologies were used, such as the eco-scale [32], the Analytical Greenness metric (AGREE) [33], and the Blue Application Grade Index (BAGI) [34]. Additionally, a thorough analysis of the many aspects of method sustainability was conducted using the RGB model [35]. These resources made it easier to comprehend the method’s overall eco-friendliness by offering a comprehensive assessment of its Whiteness (sustainable) and Greenness (environmental effect) at different phases.

Before practical laboratory application, it is essential to ensure that analytical procedures are ecologically sustainable in order to minimize chemical dangers. Furthermore, it is strongly advised that method validation include a variety of greenness evaluation techniques. Since every assessment model has drawbacks, using many models at once provides a comprehensive assessment by utilizing their unique features to enhance one another. The proposed method was thoroughly evaluated using the RGB multi-criteria model and WAC scores, taking into account economic factors, Green analytical chemistry (GAC) principles, and validation criteria [61–63].

### Red model-based scoring for validation efficiency

The validation efficiency of the published and proposed HPLC methods was evaluated using the four Red model principles (R1–R4; Table 5) in accordance with ICH guidelines. The proposed method scored higher due to its multi-analyte determination of COL and DEX in both pharmaceutical and biological samples under the same optimized chromatographic conditions with acceptable sensitivity, achieving a total score of 98.8/100 in the Red model validation assessment.

**Table 5:**
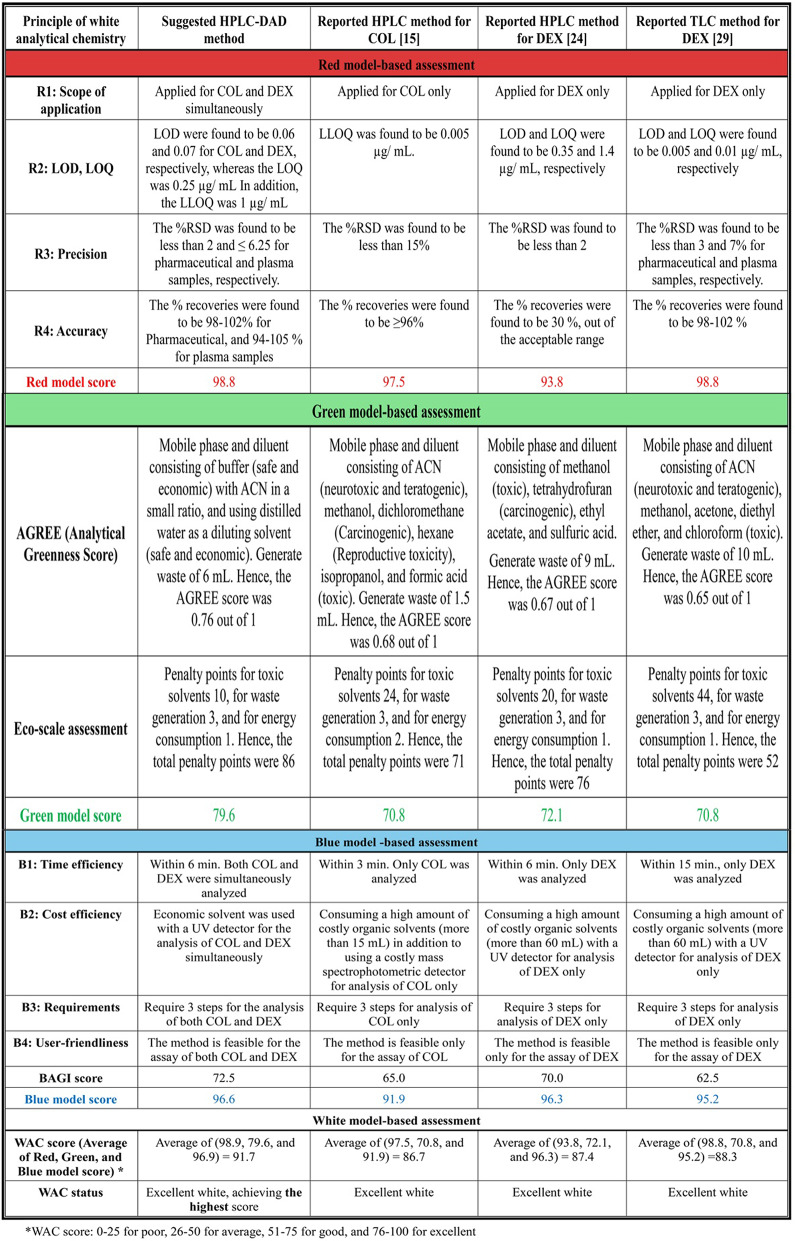
WAC-based assessment of reported and suggested HPLC method for Estimation of COL and DEX using RGB model, AGREE calculator, analytical Eco-scale, and BAGI software

### Green model-based scoring for the greenness of the procedure

The Green model assessment was conducted based on four core GAC principles (G1–G4; Table 5), using various GAC evaluation tools, including the Analytical Eco-Scale and the AGREE calculator software. The reported methods relied heavily on organic solvents such as acetonitrile, methanol, acetone, hexane, ethyl acetate, and dichloromethane in the mobile phase and exhibited longer retention times, generating more waste and increasing environmental hazards. As a result, these methods scored 70.8, 72.1, and 70 out of 100, respectively. In contrast, the proposed simultaneous HPLC analysis of COL and DEX featured a shorter run time and generated only 6 mL of waste per run, with a lower proportion of organic solvents, thereby reducing environmental impact. Consequently, our method achieved a higher Green score of 79.6/100.

Besides, the greenness of analytical methodologies was assessed using the Analytical Eco-Scale. Its foundation is the imposition of penalty points on analytical process parameters that deviate from the optimal green analysis. Different parameters and analytical processes are compared using this method. As a substitute for conventional green chemistry measures, the analytical Eco-Scale is regarded as a useful, semi-quantitative instrument, as described in Table S3. Our method has deservedly achieved the highest penalty points 86.

Key benefits of the AGREE calculator include automation, in-depth insights, and the ability to identify shortcomings in analytical methodologies that may require further environmental improvements. AGREE offers more automation and simplicity than GAPI, allowing for accurate and efficient evaluations of the greenness of analytical methods. The clock-like graph illustrating the AGREE results effectively highlights the SIGNIFICANCE mnemonic of GAC. Notably, our method achieved a higher AGREE score of 0.76 (Table 6).

**Table 6:**
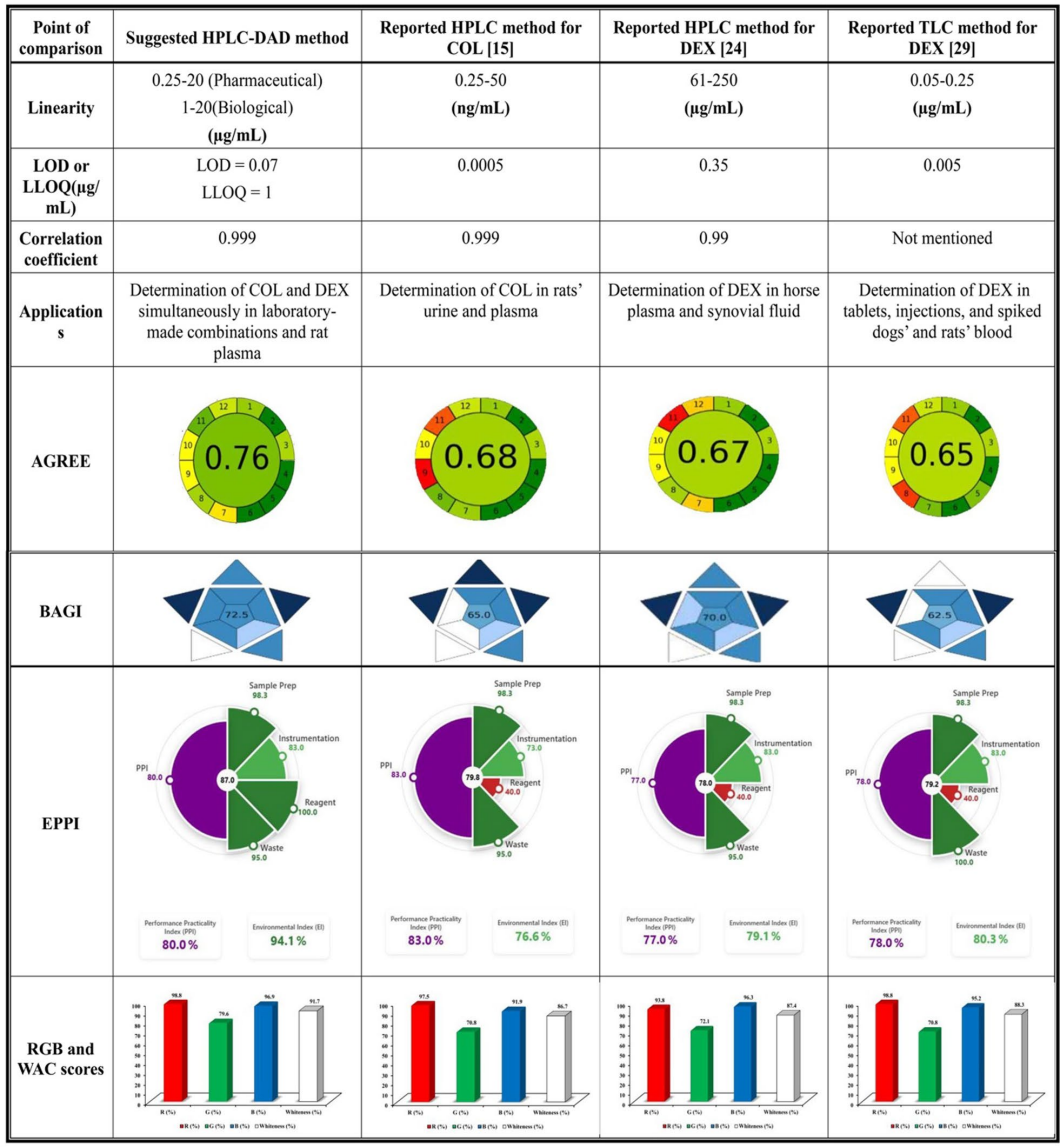
Comparison of the suggested HPLC method for the determination of COL and DEX with other reported methods

#### Blue model-based scoring for cost and time efficiency

The Blue model–based assessment was performed using four blue principles (B1–B4; Table 5) in conjunction with BAGI software to compare the cost and time efficiency, and user-friendliness of the reported and proposed methods. The reported methods for COL and DEX determination relied on costly organic solvents, particularly acetonitrile, methanol, ethyl acetate and hexane, used in higher proportions than in the proposed method. In contrast, the suggested method employs acetonitrile in a minimal proportion within the mobile phase (40% ACN: 60% buffer), rendering it safer and more economical than the organic-solvent-intensive reported methods. Additionally, the proposed method utilizes distilled water as a diluent and a UV detector rather than expensive mass-spectrometric detection, further reducing operational cost and complexity. Owing to its shorter analysis time, lower solvent consumption, reduced cost, and enhanced user-friendliness, the proposed method achieved a superior Blue model score of 96.9 out of 100.

This is further supported by the use of the cutting-edge BAGI software, which provides a straightforward approach to evaluating the effectiveness and feasibility of an analytical method in terms of blueness. It effectively highlights the method’s applications, usability strengths, and areas for further development. The suggested approach scored 72.5 on the BAGI evaluation, placing it in the middle of the asteroid pictogram (> 60 points), indicating great practicality (Table 6).

#### Principles of white analytical chemistry and method assessment

White color results from the integration of the red, green, and blue components; accordingly, the WAC scores of the reported and proposed methods were determined by averaging the scores obtained from the red, green, and blue models (Table 5). The reported chromatographic methods for the determination of COL or DEX achieved WAC scores of 86.7, 87.4, and 88.3 out of 100, respectively. In comparison, the proposed HPLC method attained a higher WAC score of 91.7/100 for the simultaneous estimation of the target drugs, indicating its excellent whiteness performance for the intended sample analysis.

**Fig. 5:**
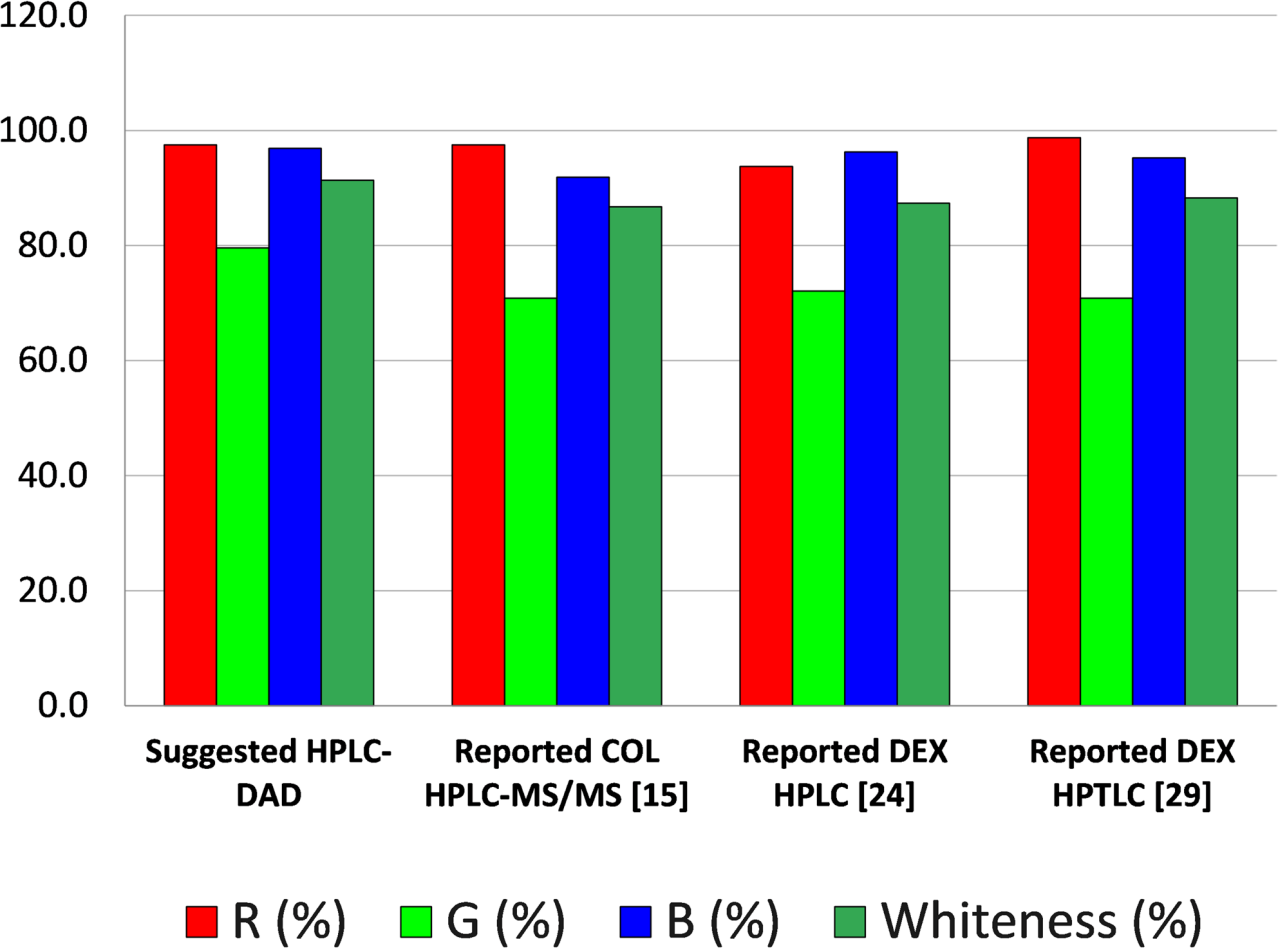
The suggested HPLC-DAD method’s RGB profiles in comparison to other reported chromatographic methods [15, 24, 29]

As shown in Table 5; Fig. 5, the suggested method achieved the highest blueness score of 96.6, along with an acceptable greenness score of 79.6 and redness score of 98.8. Additionally, it attained the highest whiteness score with an arithmetic mean of 91.7, outperforming previously reported methods [15, 24, 29].

The high whiteness score obtained through the RGB model can be attributed to the fact that the previously reported chromatographic methods [15, 24, 29] had extended analysis times (8 min, 15 min), leading to increased reagent consumption, waste generation, and reliance on HPLC-MS technology, which has the drawbacks of being high cost and energy consumption. In contrast, the suggested HPLC-DAD method offers a shorter analysis time (6 min), reduced reagent usage, minimal waste production, and lower energy consumption. Furthermore, the high redness score is indicative of the method’s complete validation, demonstrating excellent accuracy, precision, and high sensitivity, particularly with lower LOD and LOQ values compared to the existing HPLC method [24].

In summary, the RGB model and WAC scores align with the fundamental goals of analytical chemistry while evaluating a variety of criteria, such as validation requirements, economic and practical considerations. Based on this methodology, the suggested HPLC-DAD method outperformed other methods for determining COL and DEX in their laboratory-made combinations. It was a very effective and sustainable analytical technique as it scored well on the greenness scale and was excellent in terms of whiteness and applicability.

### Environmental, performance, and practicality index

The EPPI (Environmental, Performance, and Practicality Index) framework employs a dual-index approach comprising the Environmental Impact (EI) Index and the Performance and Practicality Index (PPI). Collectively, these complementary indices enable a comprehensive assessment of analytical methods by concurrently considering environmental sustainability, analytical efficiency, and real-world applicability. The EI Index is based on the combined principles of GAC and Green Sample Preparation and evaluates method greenness across all analytical stages, including pre-analytical processes, sample preparation, and instrumental analysis. In parallel, the PPI evaluates method performance and practicality by incorporating analytical effectiveness (redness) and operational applicability (blueness). EPPI outcomes are reported as a quantitative score ranging from 1 to 100 and are additionally presented as a visual pie chart, in which green reflects environmental impact and purple represents the combined contribution of performance and practicality. To enhance objectivity and reproducibility, predefined scoring criteria are applied to all assessment parameters, facilitating unbiased comparison among different analytical approaches [37]. As shown in Table 6, the proposed HPLC method exhibited enhanced sustainability, achieving a higher EPPI score (87) than reported methods due to the use of a minimum amount of organic solvent instead of a high amount of toxic organic solvents such as acetonitrile, methanol, acetone, hexane, ethyl acetate, and dichloromethane. It also allows for the simultaneous determination of COL and DEX using a UV detector, which requires lower energy than the MS-based method [15], and provides higher sensitivity by enabling microgram-level quantification rather than milligram-level quantification used in the reported method [24].

### Violet innovation grade index (VIGI) assessment

VIGI is a cutting-edge tool for evaluating the degree of innovation in analytical methods. Unlike traditional green metrics, which primarily focus on environmental sustainability. VIGI evaluates the creative potential of analytical methodologies, bridging the gap between green chemistry concepts and practical application. It adds a holistic assessment that improves the existing red, green, and blue metrics. It is based on ten criteria that assess whether a method employs advanced sample-preparation strategies, integrates novel data-processing techniques, and considers modern analytical chemistry principles, metrics, or indexes. VIGI also examines the extent to which the method addresses recognized analytical needs or recommendations from relevant organizations or regulatory bodies, utilizes innovative materials or reagents, and incorporates miniaturized or automated analytical devices. Furthermore, the index evaluates the method’s applicability across different scientific or industrial fields, improvements in sensitivity through enhanced LOD and LOQ, and the introduction of new analytical approaches. Together, these criteria provide a structured and comprehensive assessment of methodological innovation. The outcome of the VIGI assessment is presented as a violet, star-shaped decagonal pictogram. The depth of the violet color reflects the level of innovation, with darker shades corresponding to higher innovation in the analytical method. In addition, a numerical value displayed at the center of the pictogram represents the overall VIGI score on a 0–100 scale, where lower scores indicate a lower degree of methodological innovation [36]. Table S4 in the supplementary material file describes the main features and criteria of the VIGI assessment.

**Fig. 6:**
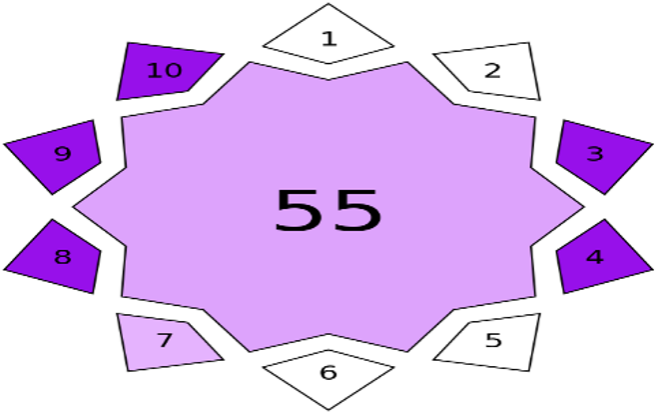
Violet Innovation Grade Index pictogram

Following the application of this survey-based method to the proposed HPLC method, Fig. 6 displays a star-shaped, decagonal pictogram with a score of 55, indicating that the suggested method is innovative (50 or above). This score is due to the incorporation of analytical chemistry matrices (green, blue, and white assessments) in our proposed method (Third parameter: white analytical chemistry and its derivatives). Furthermore, the recommended methodology adheres to ICH and FDA guidelines (Fourth parameter: Regulatory compliance). The technique is also regarded as semiautomated due to the use of an autosampler (Seventh parameter: Automation grade). Moreover, the proposed method can be used in routine analysis in quality control laboratories, bioanalytical studies, future pharmacokinetic and clinical research, supporting the therapeutic value of the COL-DEX combination, besides fostering partnerships between researchers from different fields, encouraging the sharing of experiences and knowledge (Eighth parameter: interdisciplinarity). The LOD/LOQ values showed significant sensitivity, reaching levels on the order of µg/mL. Detecting extremely low analyte concentrations in complex biological samples demonstrated the method’s effectiveness and accuracy (Nineth parameter: Sensitivity). Finally, this work represents the first chromatographic approach for concurrently analyzing COL and DEX, paving the way for future pharmacokinetic investigations and clinical studies of this promising dual-drug regimen in multi-disease management (Tenth parameter: Approach).

Ultimately, after calculating the results for each parameter, the study’s analytical approach scored 55 points. This result indicates that the approach showed a considerable level of innovation, particularly in crucial areas such as considering analytical chemistry principles, alignment with global regulatory frameworks, automation, interdisciplinary, high sensitivity (low LOD/LOQ), and novel approaches.

### Comparison with reported methods

The advantages of the proposed HPLC method over reported methods arise from a balanced integration of conventional analytical performance and GAC principles. Analytically, the depicted method enables the simultaneous, selective, and sensitive determination of COL and DEX without matrix interference, whereas most reported methods are limited to the determination of a single drug. The proposed method exhibits a broad linearity range and a low LOD, demonstrating high sensitivity compared with the reported HPLC method [24]. Additionally, the method was successfully applied to both laboratory-made combinations and rat plasma, unlike the reported TLC method, which was validated only using spiked biological samples [29].

From a greenness perspective, the method achieved high scores across multiple sustainability assessment tools (Analytical Eco-Scale, AGREE, BAGI, EPPI, and WAC), as summarized in Tables 5 and 6. This enhanced sustainability performance is mainly attributed to the high aqueous content of the mobile phase, reduced energy consumption, and reliance on UV detection rather than a mass spectrometric detector, as reported in the literature [15]. In contrast, the reported methods employ hazardous organic solvents associated with environmental and occupational risks, such as dichloromethane, hexane, acetone, chloroform, and tetrahydrofuran. Overall, the proposed method demonstrates strong analytical performance combined with improved environmental sustainability.

## Conclusion

In this study, a novel and eco-friendly HPLC-DAD method was successfully developed and validated for the simultaneous determination of COL and DEX in laboratory-made combinations and rat plasma. The method proved suitable for routine quality control and preclinical bioanalytical applications, offering a short run time (≤ 6 min), high sensitivity (LOQ = 0.25 µg/mL; LOD = 0.06–0.07 µg/mL), robust extraction recoveries (> 85%), and excellent linearity (*r* > 0.999). Its applicability was further demonstrated through effective analysis of biological samples obtained following different administration routes. The environmental sustainability of the proposed method was rigorously verified using multiple greenness assessment tools, confirming its compliance with contemporary GAC principles. To the best of our knowledge, this is the first chromatographic method enabling the concurrent quantification of COL and DEX in both pharmaceutical and plasma matrices, and among the few to incorporate a comprehensive, multi-tool sustainability evaluation, including VIGI and EPPI. Accordingly, the method represents a reliable and sustainable alternative for routine quality-control laboratories and provides a solid analytical platform for future pharmacokinetic and clinical investigations of this dual-drug regimen.

## Supplementary Information

Below is the link to the electronic supplementary material.

Supplementary Material 1

## Data Availability

All data generated or analyzed during this study are included in this published article [and its supplementary information files].

## Abbreviations

EPPI: Environmental, Performance, and Practicality Index
FDA: Food and Drug Administration
FMF: Familial Mediterranean fever
HQC: High-quality control
ICH: International conference on harmonization
IP: Intraperitoneal
IS: Internal standard
LLOQ: Low limit of quantitation
LOD: Limit of detection
LOQ: Limit of quantitation
MQC: Medium quality control
QC: Quality control
ULOQ: Upper limit of quantitation
VIGI: Violet Innovation Grade Index
