# Supplementary material for: Sustainability-based assessment using HPLC method for estimation of colchicine and dexamethasone for multi-disease treatment: application to laboratory-made combination and rat plasma
Source: BMC Chem. 2026 Mar 16;20(1):77. doi: 10.1186/s13065-026-01723-2 (PMC13063439; doi:10.1186/s13065-026-01723-2)
Supplement: Supplementary file 1 — Supplementary Material 1 [file 13065_2026_1723_MOESM1_ESM.docx]

|  | **** |
| --- | --- |
|  | |

Figure S1: The Effect of the mobile phase's percent acetonitrile on the (a) retention time, (b) asymmetrical factor

**Figure S2: Impact of various solvents on the effectiveness of COL and DEX extraction from plasma**

**(a)**

**
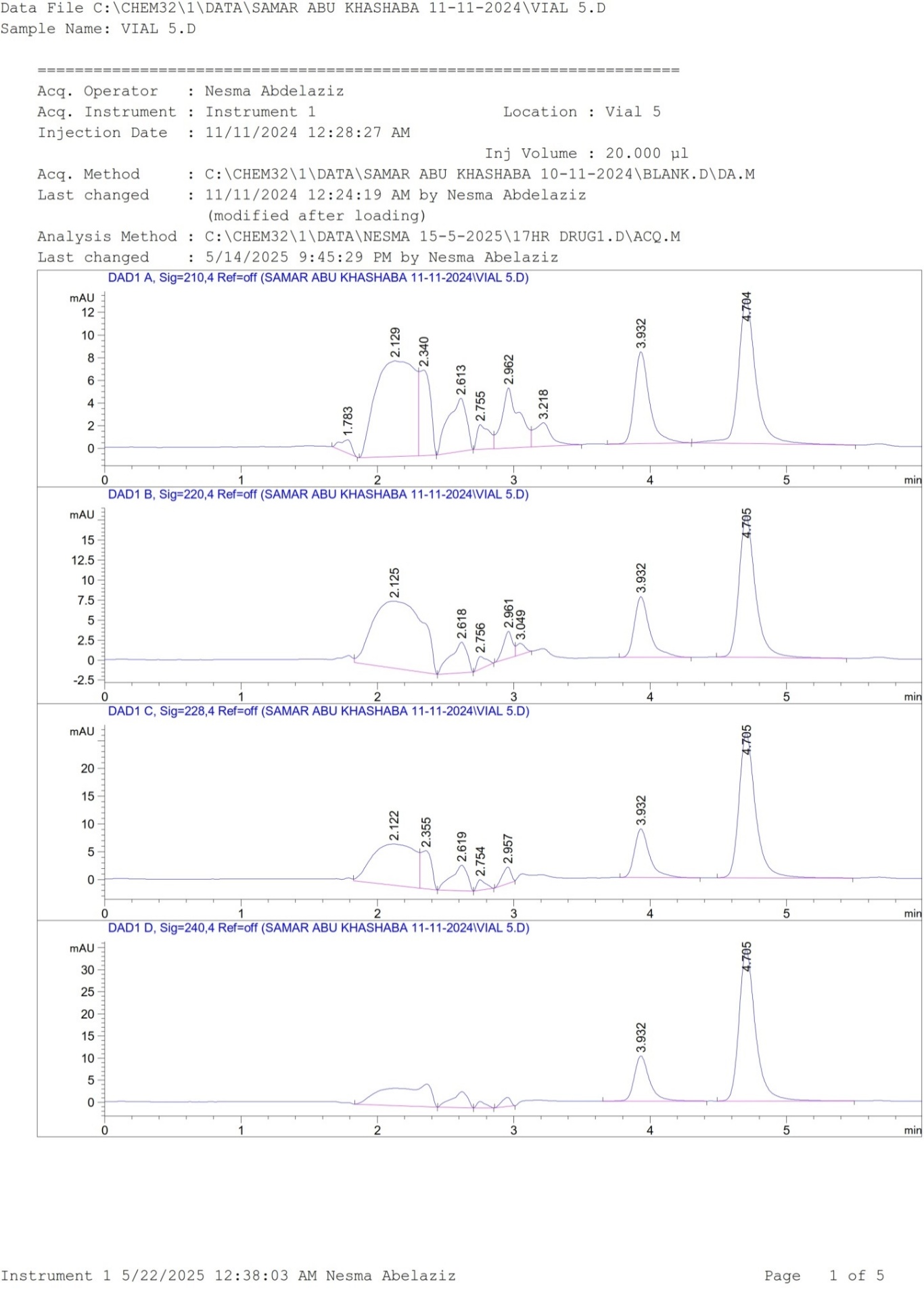
**

**(b)**

| **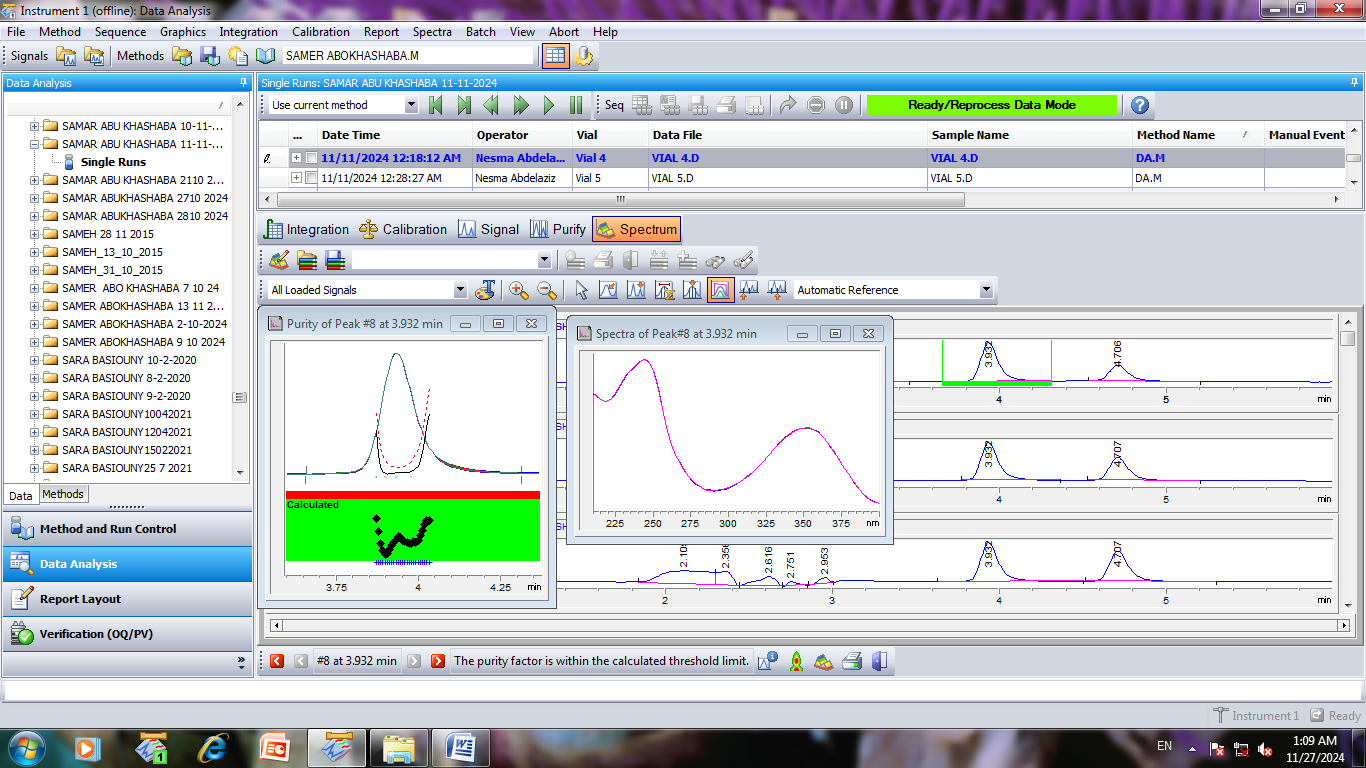** | **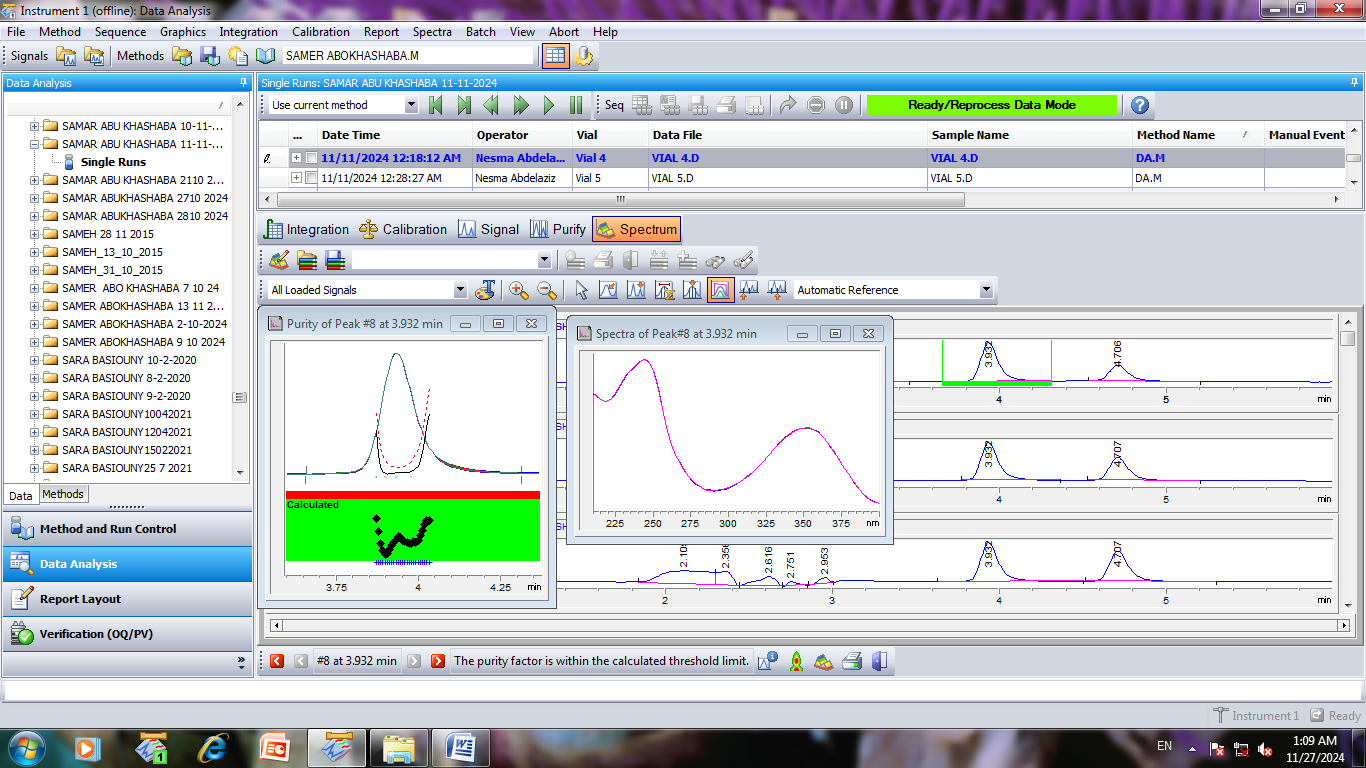** |
| --- | --- |
| **(c)** | |
| 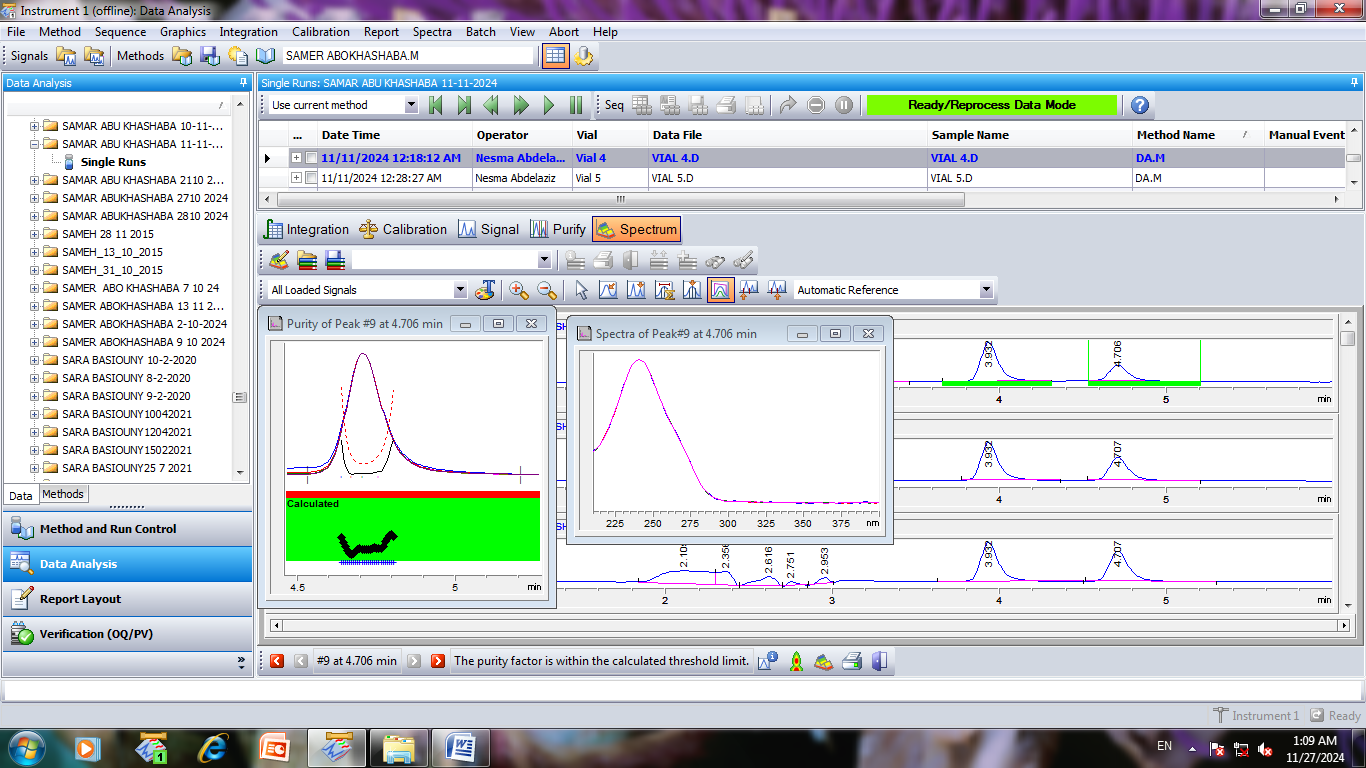 | 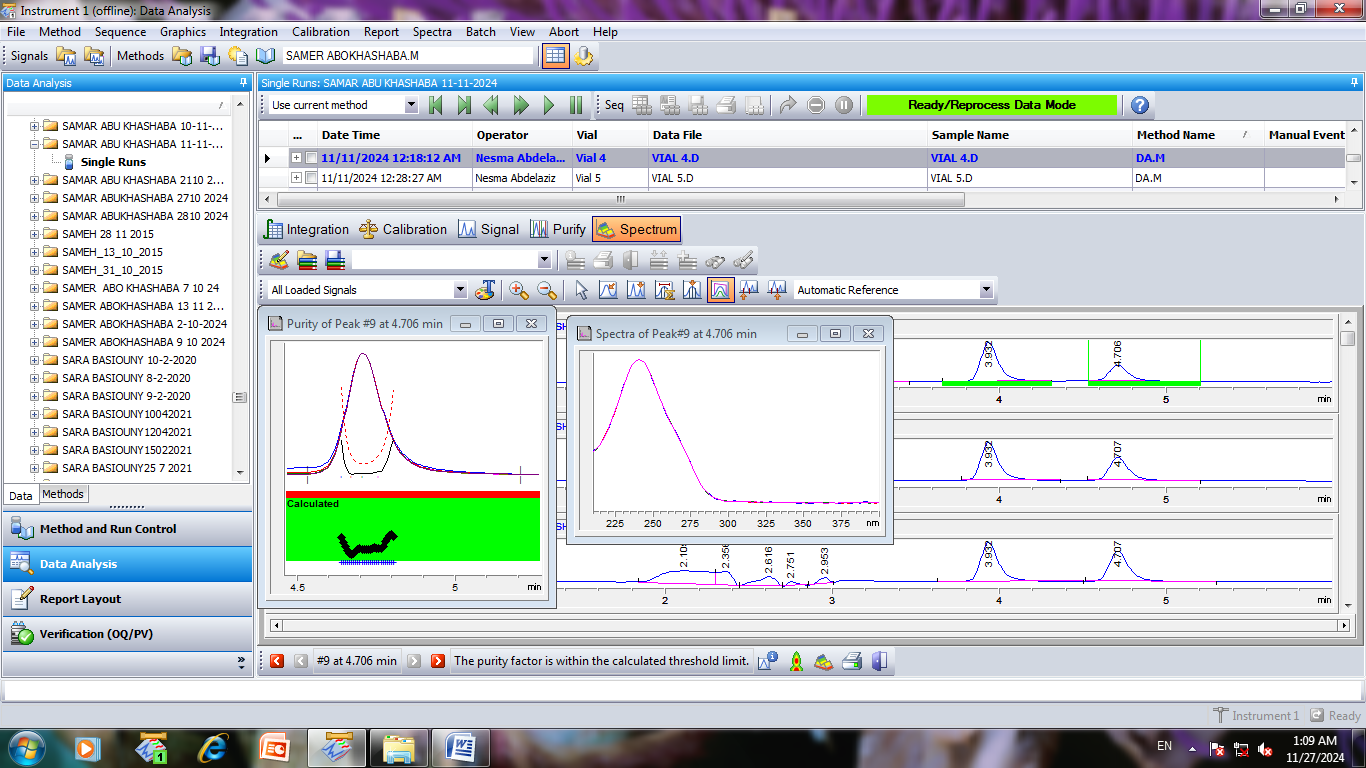 |

**Figure S3: Chromatogram of COL 1 µg/mL and DEX 5** **µg/mL in a laboratory-made combination (a) Purity profiles and plots obtained for COL (b) at 3.9 minutes and DEX (c) at 4.7 minutes**

**(a)**

| **** | **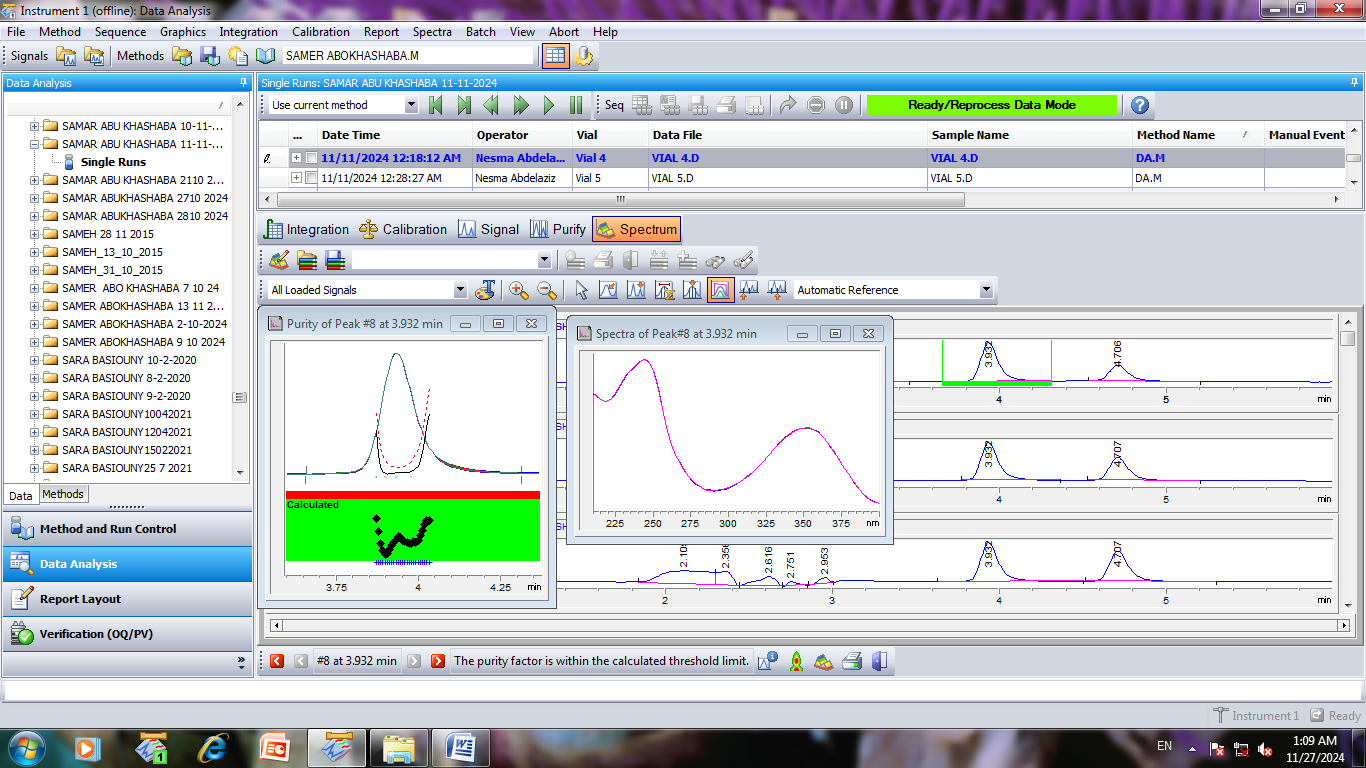** |
| --- | --- |
| **(b)** | |
| 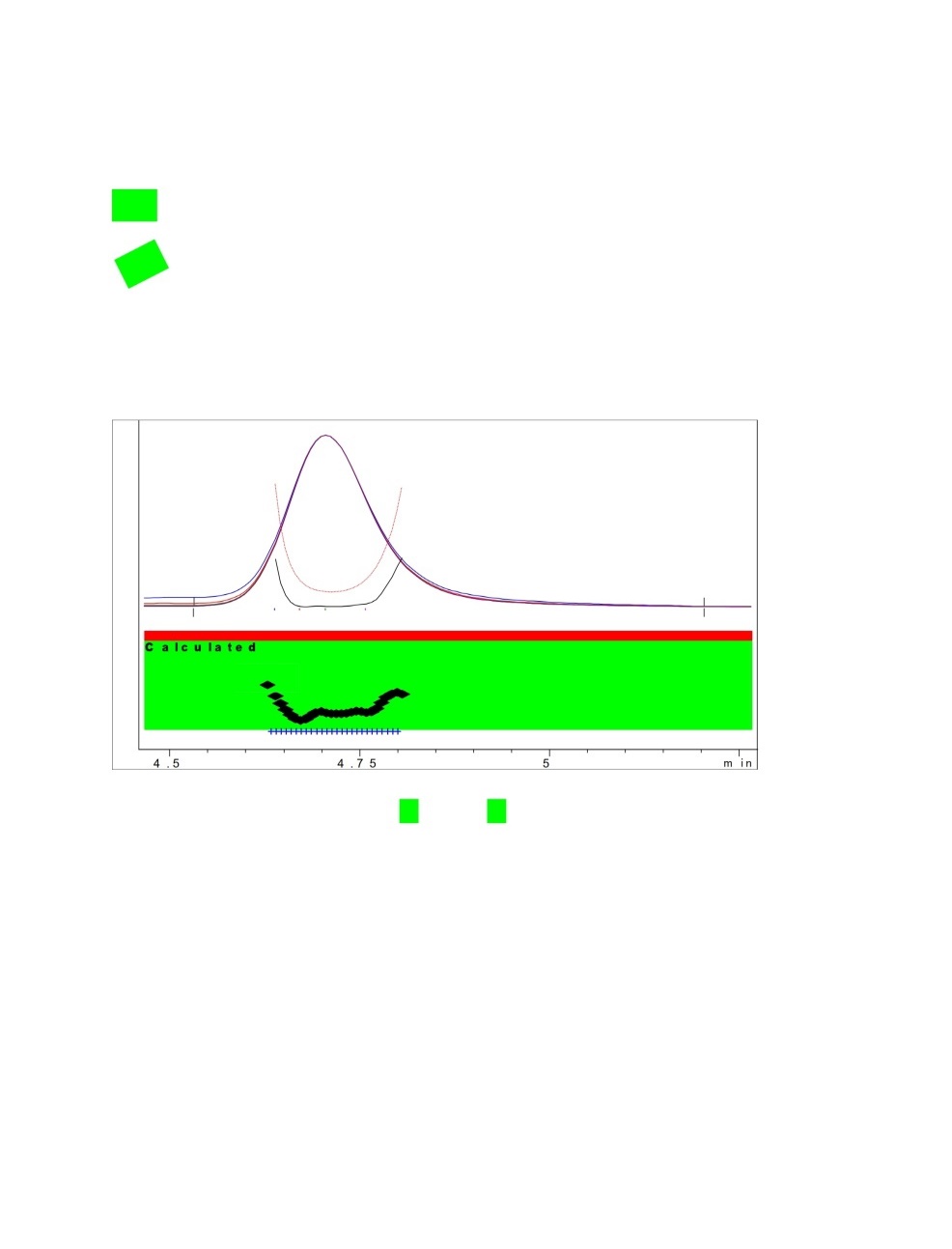 |  |
| **(c)** | |
| 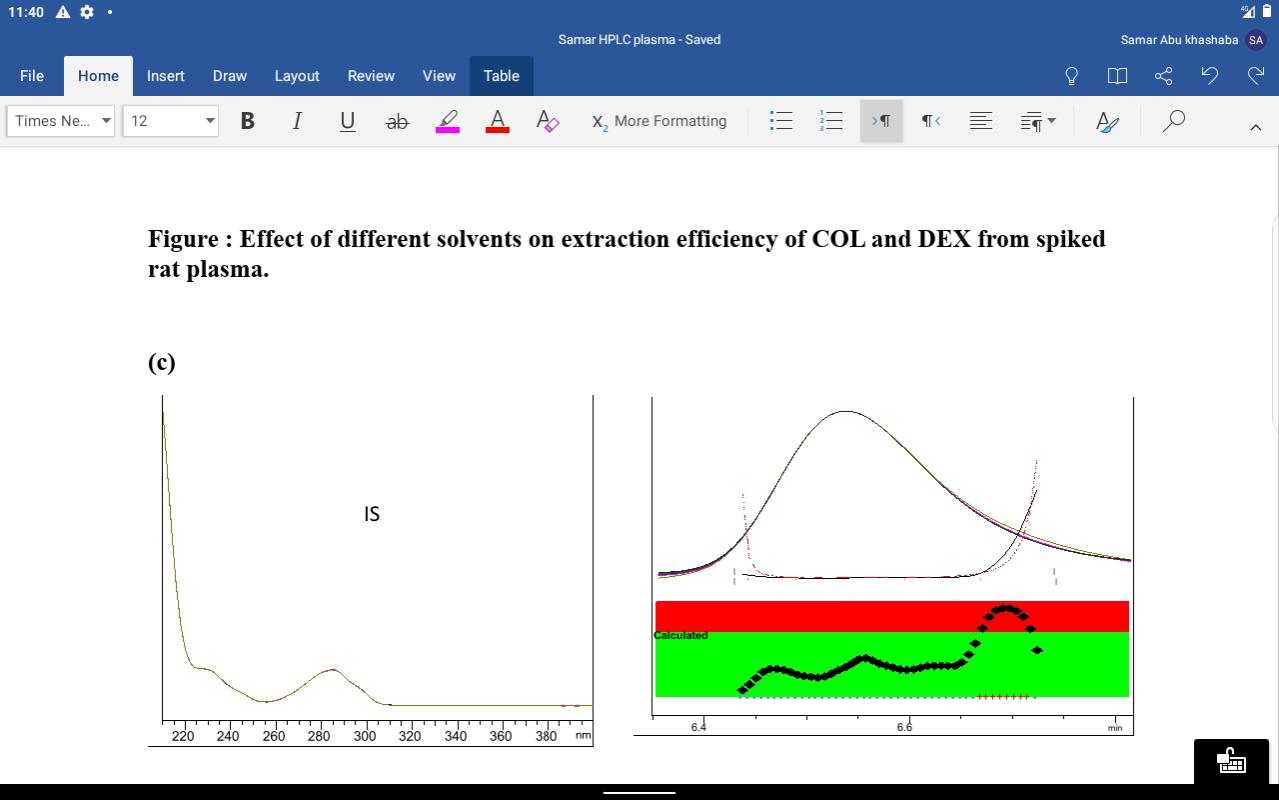 | |
|  | |

Figure S4: Superimposed absorption spectra and purity profile illustrating peak purity of (a) COL, (b) DEX, and (c) IS obtained from spiked plasma extracted from DAD

Table S1: Using the suggested HPLC-DAD approach, a stability overview of the medications under study in spiked rat plasma samples at two concentration levels (LQC and HQC) (n = 6)

| Stability conditions | | COL (µg/mL) | | DEX (µg/mL) | |
| --- | --- | --- | --- | --- | --- |
|  |  | **3** | **15** | **3** | **15** |
| Freeze and Thaw  Stability  After 5 cycles at - 20°C | **Recovery%** | 107.41 | 104.89 | 96.01 | 103.99 |
|  | **RSD%** | 4.57 | 2.71 | 4.59 | 2.91 |
| Post preparative  (5°C for 24 h) | **Recovery%** | 105.02 | 90.04 | 103.21 | 105.67 |
|  | **RSD%** | 6.81 | 7.71 | 4.78 | 4.72 |
| Long-Term  Stability  20 days at -20°C | **Recovery%** | 108.23 | 92.21 | 93.98 | 94.01 |
|  | **RSD%** | 6.56 | 3.09 | 4.02 | 4.21 |
| Bench-Top  Stability(6h) | **Recovery%** | 105.02 | 94.34 | 92.56 | 96.49 |
|  | **RSD%** | 5.41 | 7.01 | 3.51 | 3.09 |

**Table S2: Results of assays for determining COL and DEX in laboratory-made combination**

| ***Laboratory-made combinations (µg/mL)** | | **Mean %recovery +SD ^a^** | | **%RSD ^b^** | | | **%Er ^c^** | |
| --- | --- | --- | --- | --- | --- | --- | --- | --- |
| **COL** | **DEX** | **COL** | **DEX** | **COL** | **DEX** | | **COL** | **DEX** |
| **1** | **5** | 99.08±0.79 | 99.96±0.81 | 0.80 | | 0.81 | -0.92 | -0.04 |
| **2** | **1** | 99.31±0.47 | 99.28±0.47 | 0.48 | | 0.47 | -0.69 | -0.72 |
| **1** | **10** | 99.12±0.78 | 99.98±0.95 | 0.79 | | 0.95 | -0.88 | -0.02 |
| **10** | **10** | 99.38±0.59 | 99.62±0.37 | 0.59 0.37 | | | -0.62 | -0.38 |

* The ratios of laboratory-made combinations are 1:5, 2:1, 1:10, 10:10 of COL and DEX respectively.

^a^ Mean recovery of the found concentration ± standard deviation for three determinations(n=3).

^b^ % Relative standard deviation.

^c^ % Relative error

**Table S3: Analytical Eco-scale for assessment of greenness of the suggested HPLC-DAD method compared with other reported chromatographic methods**

| **Reagent** | **Suggested HPLC-DAD** | **Reported COL HPLC-MS/MS**  **[**[**15**](#_ENREF_15)**]** | **Reported DEX HPLC**  **[**[**24**](#_ENREF_24)**]** | **Reported DEX HPTLC [**[**29**](#_ENREF_29)**]** |
| --- | --- | --- | --- | --- |
| Acetonitrile | 4 |  |  | 8 |
| Methanol | 6 | 6 | 6 | 12 |
| Acetone |  |  |  | 4 |
| Diethyl-ether |  |  |  | 4 |
| Chloroform |  |  |  | 16 |
| Dichloromethane |  | 2 |  |  |
| Hexane |  | 8 |  |  |
| Isopropanol |  | 4 |  |  |
| Ammonium acetate |  | 0 |  |  |
| Formic acid |  | 4 |  |  |
| Triethylamine |  |  |  |  |
| Tetrahydrofuran |  |  | 6 |  |
| Sulphuric acid |  |  | 4 |  |
| Ethyl acetate |  |  | 4 |  |
| Dihydrogen phosphate | 0 |  |  |  |
| Total penalty points for the reagent | 10 | 24 | 20 | 44 |
| Energy | 1 | 2 | 1 | 1 |
| Waste | 3 | 3 | 3 | 3 |
| Occupational hazard | 0 | 0 | 0 | 0 |
| Final total penalty points (sum of reagent and instrument points) | 14 | 29 | 24 | 48 |
| Analytical eco-scale score | **86** | **71** | **76** | **52** |

|  |  |  | **Violet** | **Light violet** | **White** |
| --- | --- | --- | --- | --- | --- |
|  |  |  | **10 points** | **5 points** | **0 points** |
| **Criterion** | **Parameter** | **Question: Does the analytical method...?** | **High innovation** | **Regular innovation** | **Low innovation** |
| **1** | **Sample prep. and instrumentation** | **Apply advanced sample preparation methods and/or techniques** | **Strongly agree** | **Moderately agree** | **Disagree** |
| **2** | **Data processing and software** | **Incorporate new data processing techniques** | **Strongly agree** | **Moderately agree** | **Disagree** |
| **3** | **White analytical chemistry and its derivatives** | **Consider analytical chemistry principles, metrics or indexes (e.g., AGREE, AGREEprep, MoGAPI, BAGI, RAPI, etc.)** | **Strongly agree** | **Moderately agree** | **Disagree** |
| **4** | **Regulatory compliance** | **Address problems, needs, and/or recommendations highlighted by relevant organizations at the local, national, or international level** | **Strongly agree** | **Moderately agree** | **Disagree** |
| **5** | **Materials and reagents** | **Utilize innovative materials and/or reagents** | **Strongly agree** | **Moderately agree** | **Disagree** |
| **6** | **Miniaturization** | **Use of miniaturized devices** | **Strongly agree** | **Moderately agree** | **Disagree** |
| **7** | **Automatization grade** | **Integrate automatization** | **Strongly agree** | **Moderately agree** | **Disagree** |
| **8** | **Interdisciplinarity** | **Extrapolate to different areas of science and/or industry and promote collaborations** | **Strongly agree** | **Moderately agree** | **Disagree** |
| **9** | **Sensitivity** | **Improve LOQ and LOD values compared to previous methods** | **Strongly agree** | **Moderately agree** | **Disagree** |
| **10** | **Approach** | **Introduce a new approach for research** | **Strongly agree** | **Moderately agree** | **Disagree** |

**Table S4: Detailed Description of the Main Features and Criteria of the VIGI**
